# The roles of histone deacetylases in kidney development and disease

**DOI:** 10.1007/s10157-020-01995-5

**Published:** 2021-01-04

**Authors:** Hongbing Liu

**Affiliations:** Department of Pediatrics and The Tulane Hypertension and Renal Center of Excellence, Tulane University School of Medicine, SL-37, 1430 Tulane Avenue, New Orleans, LA 70112 USA

**Keywords:** Histone deacetylases, Histone deacetylase inhibitors, Kidney disease, Kidney cancer

## Abstract

Histone deacetylases (HDACs) are important epigenetic regulators that mediate deacetylation of both histone and non-histone proteins. HDACs, especially class I HDACs, are highly expressed in developing kidney and subject to developmental control. HDACs play an important role in kidney formation, especial nephron progenitor maintenance and differentiation. Several lines of evidence support the critical role of HDACs in the development and progression of various kidney diseases. HDAC inhibitors (HDACis) are very effective in the prevention and treatment of kidney diseases (including kidney cancer). A better understanting of the molecular mechanisms underlying the role(s) of HDACs in the pathogenesis and progression of renal disease are likely to be of great help in developing more effective and less toxic selective HDAC inhibitors and combinatorial therapeutics.

## Introduction

Epigenetics refers to the study of heritable changes in gene expression and regulation independently of DNA sequence. Recent years have witnessed an emerging awareness of the critical role of epigenetic mechanisms in health and disease [1]. During development, epigenetic modifications, such as DNA methylation, histone acetylation, and histone methylation, are set in the chromatin to determine the genome programming in a particular cell by alteration of chromatin structure and thus DNA accessibility to the transcriptional machinery. Disruptions of these epigenetic modifications resulting from environmental exposures (e.g., diet, toxins, drugs, viral infections) can lead to dysregulation of gene function, without altering the DNA sequence itself [1–3]. As epigenetic abnormalities depend on the interplay between genes and the environment, they are often phenotypically variable, which fits well with the broad phenotypic spectrum of congenital anomalies of the kidney and urinary tract (CAKUT) and other kidney disease [4–9]. Therefore, understanding the epigenetic basis of kidney development might provide new insights into the pathological mechanisms of kidney and, hopefully, open new avenues to treatment or prevention of CAKUT, through pharmaceutical agents that target epigenetic modifiers. Such epigenetic drugs are already in clinical use or under investigation for the treatment of cancer as well as other diseases [10].

Histone acetylation leads to the covalent addition of an acetyl group to a lysine. Such an addition results in local open chromatin, a mark of active transcription, by neutralizing the net positive charge of histone tail and reducing its binding to negatively charged DNA. In contrast, deacetylated histones interact strongly with DNA and result in local close chromatin, a mark of inactive transcription. Differential acetylation of promoter and enhancer histones plays a very important regulatory role in developmental processes, cellular proliferation and differentiation. Aberrant acetylation or deacetylation leads to diverse disorders such as leukemia, epithelial cancers, fragile X syndrome and Rubinstein-Taybi syndrome [11]. Histone deacetylases (HDACs) are a large family of evolutionarily conserved enzymes which catalyze the removal of acetyl groups from histone tails. The action of HDACs is counteracted by histone acetyltransferases (HATs), which acetylate histone tails. To date, 18 mammalian HDACs have been identified. According to phylogenetic analysis and their functions, HDACs are divided into four classes: class1 (Hdac1-3, and 8), class II (Hdac4-7, 9–10), class III (Sir2/Sirt 1–7), and class IV (Hdac11). Class I, II and IV require zinc for their catalyze activity, while class III members rely on NAD for activity. Among them, class I HDACs have been extensively studied and characterized. Given that histone acetylation is commonly associated with active transcription, HDACs were originally regarded as general transcriptional co-repressors. However, later on, it has become clear that HDACs regulate gene expression in a highly selective way and exhibit both repressive and activating effects [12]. Due to a lack of intrinsic DNA binding activities, HDACs cannot operate alone for their functions in various fundamental biological processes [12].

It has become clear that HDACs can act on numerous substrates in addition to histones. It is difficult to determine the histone substrate specificity of different HDACs. The main reason is that many purified HDACs possess very low deacetylation activity and functional redundancy of different HDACs [13]. The loss or knockdown of one member of HDACs may not be enough to change the overall histone acetylation. In addition, HDACs are recruited to target genes via association with transcriptional complexes (e.g. Sin3 complex, NuRD complex, and Co-REST complex) [14]. Thus, the specificity of HDACs in gene regulation depends on the partner proteins they associate within different cell types. Although three members of class I HDACs (HDAC1, HDAC2 and HDAC3) generally share similar sequence features, some studies showed that HDAC3 (and perhaps all class I HDACs) has distinct substrate specificity [15, 16]. Very likely, each HDAC has its specific target profile in certain substrates, which requires a more comprehensive investigation. Like almost all enzymes, HDACs are functionally regulated for their activities. Among numerous regulation mechanisms at different levels, protein–protein interaction and post-translation modifications (PTMs) are the two well-fined mechanisms. Many HDACs do not function alone but act as a component in a multiprotein complex, which help individual HDAC to exert its catalytic activity in a more effective and specific manner [17]. It is very common for HDACs to function through the formation of large protein complexes. Extensive biochemical analyses showed that HDAC1 and HDAC2 co-exist in three major multiprotein co-repressor complexes: Sin3, NuRD (nucleosome remodeling and deacetylation) and CoREST (co-repressor for element-1-silencing transcription factor) [14]. HDACs are not only protein modifiers, they can also be the subject to various PTMs. Phosphorylation is the most extensively studies modification. HDAC1 can be phosphorylated by casein kinase (CK2) and cAMP-dependent kinase PKA [18]. Phosphorylation at both Serine (S) 421 and S423 is essential for the enzymatic activity of HDAC1, and mutations of these two sites significantly decrease its activity and complex formation. CK2 can also phosphorylate HDAC2 on sites 422 and 424 (homologues to S421 and S423 of HDAC1), and on S394, the main phosphorylation site of HDAC2 [19]. Consistently, we also found the presence of S394 phosphorylated Hdac2 in the developing kidneys via mass spectrometry analysis (Liu et al., unpublished). In addition, phosphorylation of HDAC1 and HDAC2 is reversibly regulated by the protein phosphatase PP1 [20]. Studies also revealed that PP2A regulates the hypertrophic response by dephosphorylating S394 of HDAC2 in the human heart [21]. Collectively, HDACs are important epigenetic modulators and play roles in a multitude of biological processes. Their activities are tightly controlled by multiple mechanisms, such as protein–protein interaction, and PTMs. The formation of a multiprotein complex determines not only the activity of HDACs but also their substrate specificity. The molecular changes by HDACs are likely to exert a significant impact on human health and disease. In this review, we would like to descript the roles of HDACs in kidney development and disease.

## The roles of HDACs in the kidney development

**Fig. 1:**
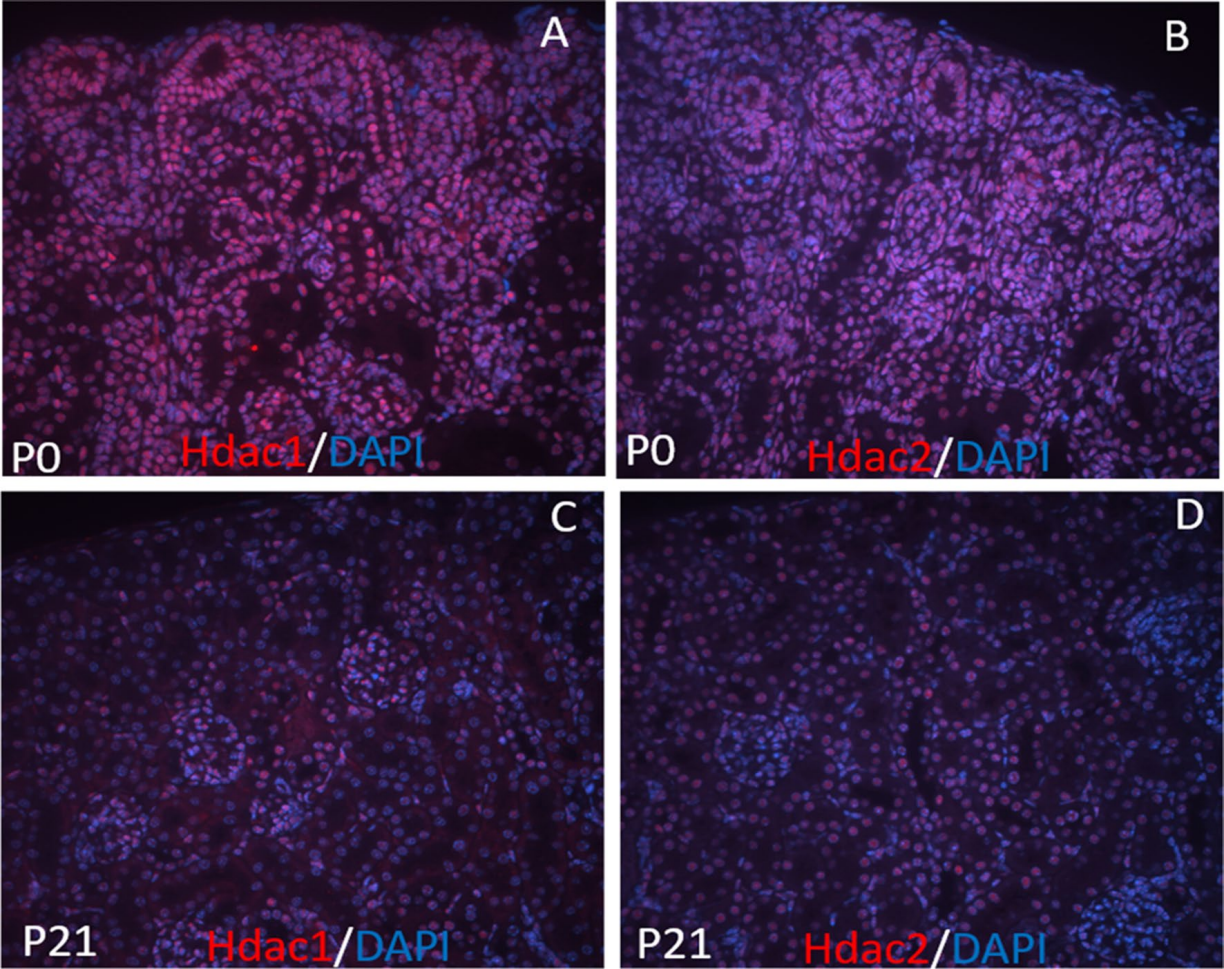
High expression and nuclear localization of Hdac1 and Hdac2 in the kidneys of new-born (P0) (**a**, **b**) and P21 (**c**, **d**) mice, respectively

The ubiquitous expression and high deacetylase activity of class I HDAC are consistent with their functional significance. HDAC1 conventional knock out mice is embryonic lethal, resulting in severe proliferation defects and growth retardation [22]. Surprisingly, the conditional knock out of HDAC1 is well tolerated in multiple tissues and the mice are viable, very likely due to the functional redundancy of HDAC1 and HDAC2 in later development and postnatal life [22]. Co-deletion of HDAC 1 and HDAC2 is detrimental in all tissues examined [22]. Moreover, HDAC2-null mice die after birth within 24 h due to heart dysfunction [22]. In the kidney, HDACs are ubiquitously and highly expressed. RT-PCR analysis indicates that *HDAC1, 2, 3, 4, 7, 9* are subject to developmental control and decline significantly during the maturation from embryonic to neonatal and adult life [23]. Western blot analysis of kidney nuclear lysates further confirms that Hdac1–3 proteins are highly abundant in the embryonic kidney and are down-regulated postnatally [23]. Immunostaining reveals that Hdac 1 and 2 are highly expressed in different populations of the developing kidney at kidneys of new-born (P0) mice, including the undifferentiated metanephric mesenchyme, branching ureteric buds, and the stroma (Fig. 1). Hdac1 and 2 are overlappingly and exclusively in nuclear expressed in developing and P21 kidneys (Fig. 1). High expression of Hdac3 was also detected in developing kidney, including podocytes. By contrast, Hdac 5, 6, and 8 are constitutively expressed. The renal microvasculature expresses Hdac 7, 8, and 9 [24]. Collectively, class I and class II *HDAC* genes are differentially regulated during kidney development. The expression of all the HDACs in the kidney was well documented by a recent review [25]. However, the spatially and temporally restricted distribution of HDACs do not change the global acetylation levels of histones H3 and H4, suggesting a tight coupling of HAT and HDAC functions during normal development [23].

Among class I HDACs, HDAC1 and HDAC2 are evolutionarily related to each other and both are very abundantly expressed in many tissues. HDAC1 and HDAC2 can form homo- or heterodimers and are found together in almost all nuclear protein complexes, including three well-characterized co-repressor complexes: Sin3, NuRD and Co-REST [14, 26]. By conditional deletion of Hdac1 and Hdac2 in ureteric bud (UB) lineage with Hoxb7-Cre, studies revealed that mice with no more than three deleted alleles of *Hdac1* and *Hdac2* exhibit no significant abnormalities in kidney development [27]. These mice can survive to adulthood without any abnormalities in growth or development. By contrast, concurrent deletion of all four alleles of *Hdac1* and *Hdac2* results in early postnatal lethality by 2–4 weeks of age [27]. The kidney tissue of knockout mice at P0 showed the absence of nephrogenic zone, lack of cortical-medullary patterning, and the formation of multiple epithelial cysts, suggesting a critical role and functional redundancy of Hdac1 and Hdac2 in kidney formation and function [27]. Studies also demonstrate that loss of Hdac1 and Hdac2 in the UB epithelium leads to marked hyperacetylation of the tumor suppressor protein p53 to boost p53 stability and transcriptional activity [27]. While p53 is a very important tumor suppressor, unconstrained p53 activity is detrimental to kidney formation, most likely due to inappropriate cellular death or proliferation defects [28].

**Fig. 2:**
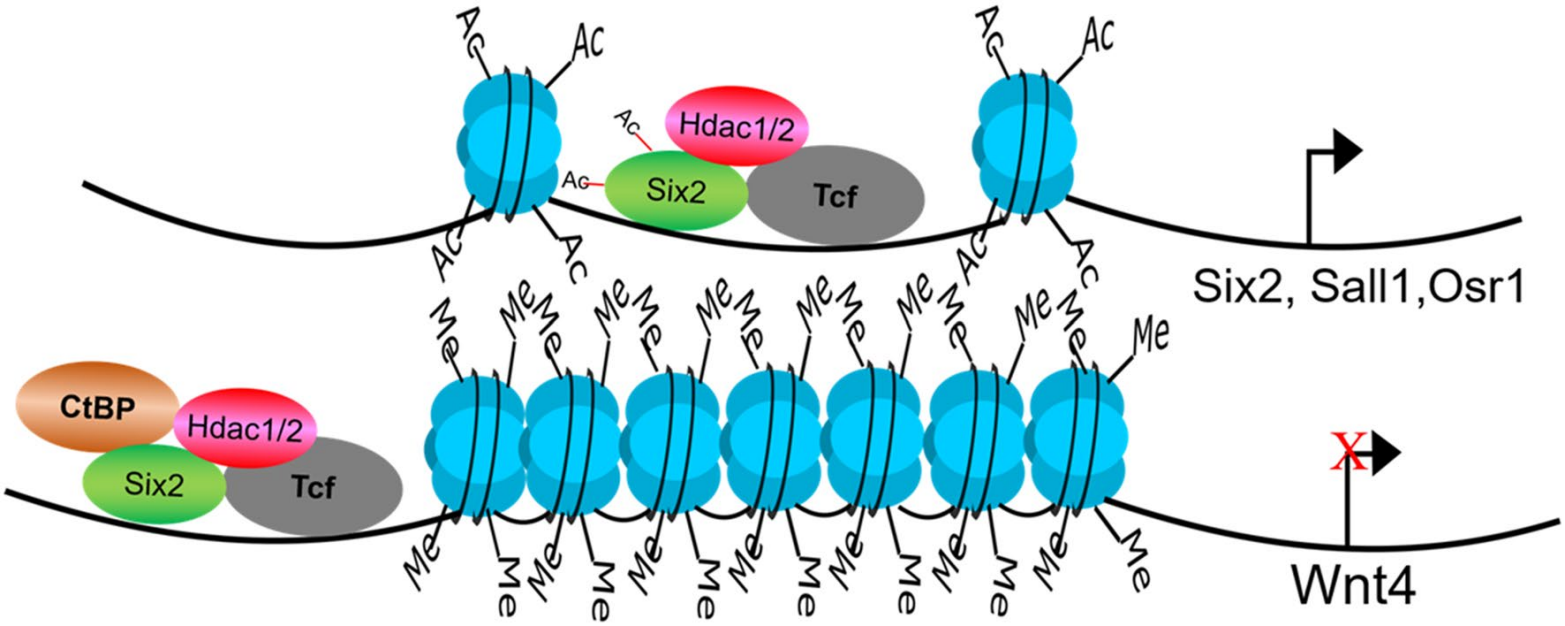
HDAC1/2 play dual roles to balance the self-renewal and differentiation of NPC during nephrogenesis

The kidney contains multiple specialized cell types with district physiological functions. Kidney formation in mammals is initiated by reciprocal interactions between two tissues types, the ureteric bud and metanephric mesenchyme [5, 29–31]. The *Six2* + cap mesenchyme is a multipotent self-renewing nephron progenitor cells (NPCs) that gives rise to the functional nephron epithelium [30]. To gain insights into the role of HDAC1/2 in NPC maintenance and differentiation, we conditionally removed Hdac1 and Hdac2 with Six2eGFPCre (Six2TGC) mice [30]. Mice with NPC-specific double deletion of HDAC1 and HDAC2 (all four alleles) were born in normal Mendelian ratios but died soon after birth [4]. The mutant kidneys at postnatal day (P) 0 showed small kidney size, absence of the nephrogenic zone, lack of nascent nephrons and glomeruli, and formation of multiple cysts [4]. Similar to the Hdac1 and Hdac2 deletion in UB lineage, one allele of either HDAC1 or HDAC2 is sufficient to ensure nephrogenesis [4]. Our results showed that histone deacetylases 1 and 2 (HDAC1/2) are essential to regulate the transcriptional programs of nephron progenitors and renal vesicles [4]. HDAC1/2 play dual roles to balance the self-renewal and differentiation of NPC during nephrogenesis (Fig. 2): On the one hand, HDAC1/2 are required for the expression of all the marker genes (such as *Six2*, S*all1* and *Osr1*) in NPC and the self-renewal of these cells; on the other hand, they are also important to repress the expression of canonical Wnt target genes and prevent the NPCs from pre-mature differentiation. Our biochemical and ChIP analyses also revealed that HDAC1 and HDAC2 interact with Six2, Osr1 and Sall1, a network of transcriptional regulators that maintain the balance of NPC proliferation and differentiation [4]. Six2 is a master transcription factor in the developing kidney and plays a central role in maintaining a functional pool of self-renewing, by dually suppressing NPCs differentiation and driving self-renewal. Six2 and HDAC1/2 are co-expressed in the undifferentiated NPCs and all are required for maintenance of the nephron progenitor cells and prevention of premature differentiation [4]. We reason that HDAC1/2 are required for Six2′s dual function to precisely control the self-renewal and differentiation of NPCs. HDACs can deacetylate not only histone proteins but also non-histone proteins [12]. The in silico prediction by the acetylation set enrichment based (ASEB) computer program [32] indicates several potential sites responsible for Six2 acetylation by p300 and deacetylation by Hdac1/2 (Table 1). Among them, Lysine (K) 46, K52, and K71 are located at Six domain (1–124) domain of Six2, and K138 is located at homeodomain of Six2 [33]. Studies showed that the Six domain has a much stronger tendency to nucleus accumulation and a protein consisting of the Six domain and the homeodomain was found to be exclusively at nucleus [33]. Therefore, the acetylation of these potential sites would be very likely associated with the nucleus localization of Six2 protein and/or transcriptional activity. Recently, our genome-wide analysis showed that Hdac1 and Six2 co-occupy the enhancer regions of NPC renewal genes and the binding of Hdac1 indicates the open chromatin at the promoter region of actively transcribed genes in NPC [34]. How HDAC1/2 to regulate Six2 function for kidney development definitely warrants further investigation.

**Table 1:** P value for candidate sites of acetylation by CBP/p300 and deacetylation by HDAC1/HDAC2/HDAC3

Site	Sequence	*P* value (KAT: CBP/p300)	*P* value (HDAC1/2/3)
46	LPACEHLHKNESVLKAK	1	0.7655
52	LHKNESVLKAKAVVAFH	0.971	0.9863
71	1GNFRELYKILESHQFS	0.9493	0.9912
138	GEETSYCFKEKSRSVLR	0.8574	0.9986

**Fig. 3:**
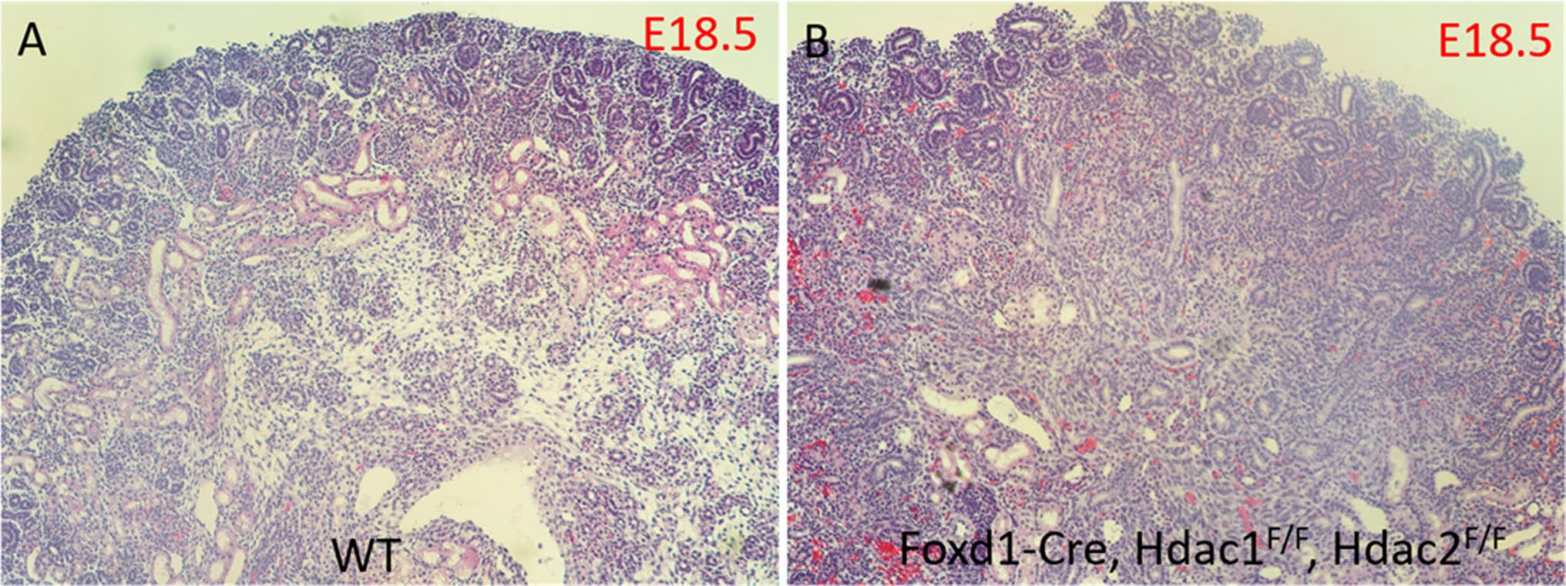
Stromal-specific deletion of Hdac1 and Hdac2 causes aberrant expansion of nephron progenitors of developing kidneys

The mammalian kidney develops from reciprocal interactions between the metanephric mesenchyme and ureteric bud. The stroma, the third lineage which fills up the interstitial space, is derived from distinct progenitors that express the transcription factor Foxd1 [35]. Studies demonstrate that the Foxd1 expressing cortical stroma is a multipotent self-renewing progenitor population, which will give rise to cortical and medullary interstitial cells, mesangial cells, and pericytes of the kidney [35]. HDAC1 and HDAC2 are also higher expressed in the stroma of developing kidney [4, 23, 27]. To examine the role of the two HDACs in stroma lineage, we are employing a mouse model to conditionally remove HDAC1 and HDAC2 with Foxd1-Cre [36]. Our preliminary results showed that specific HDAC1/2 deletion in stromal progenitors leads to aberrant expansion of nephron progenitors (Fig. 3), the similar phenotype observed in Foxd1-Cre driven *Sall1* deletion or ablation of the renal stroma [37, 38]. Further characterization on how stromal HDAC1/2 to restrict and regulate excessive nephron expansion would help advance our understanding toward the epigenetic regulation of kidney formation and the cross talk between stromal and nephron.

## HDACs and renal interstitial fibrosis

Chronic kidney disease (CKD) is an increasingly recognized public health issue and marked by a gradual and irreversible decline in kidney function. Renal interstitial fibrosis is a hallmark of CKD. Renal interstitial fibrosis is characterized by renal tubular atrophy, abnormal deposition of extracellular matrix (ECM) and progressive expansion of fibroblasts. The underlying pathogenic mechanisms are complex and diverse. Accumulating evidence has shown that HDACs participate in the pathogenies of renal interstitial fibrosis and HDAC inhibition exerts anti-fibrotic effects mainly via following mechanisms: (1) suppressing the pro-fibrotic TGF-β signaling, (2) preventing tubular epithelia cell apoptosis, and (3) increasing the expression of bone morphogenesis protein 7 (BMP7) [39]. Transforming growth factor beta (TGF-β) is a member of the transforming growth factor, which is essential in the regulation of various biological processes. In mammals, three isoforms of TGF-β have been identified in the kidney: TGF-β1, TGF-β2, and TGF-β3. Among them, TGF-β1 exerts its biological activity through Smad and non-Smad pathways and its role in renal fibrosis is the best characterized (Yu et al. 2003; Meng et al. 2016). Aberrant activation of TGF-β1 in the kidney causes interstitial fibrosis by the promoting fibroblast proliferation and deposition of abnormal extracellular matrix [40]. Earlier studies demonstrated that treatment with trichostatin A (TSA), a pan HDAC inhibitor (HDACi) for both class I and class II HDACs, attenuates renal fibrosis in unilateral ureteral obstruction (UUO) mouse model [41]. TSA treatment also significantly inactivated fibroblasts. Moreover, silencing of HDAC1 or HDAC2 blocked renal fibroblast proliferation by reducing phosphorylation of STAT3 (signal transducer and activator of transcription 3), a signaling molecule associated with the proliferation of renal fibroblasts and the development of renal fibrosis [42]. Further studies demonstrated that TSA treatment also upregulates the expression of BMP-7 and attenuates the pathogenesis of renal injury [43, 44]. As BMP-7 induces protection against TGF-β-mediated renal fibrosis, restoration of BMP-7 expression represents another major mechanism by which HDAC inhibition prevents progressive CKD [44]. In addition, administration of MS-275 or Fk228 (selective inhibitors of class I HDACs) significantly attenuate the progression of kidney fibrosis by inhibiting renal fibroblast activation and proliferation, suggesting that class I HDACs play predominant roles in renal fibrosis [45, 46].

Collectively, HDACs play important roles to renal fibroblast activation and renal interstitial fibrosis, and the use of inhibitors of HDACs (especially class I HDACs) may therefore provide effective therapy to alleviate and treat renal fibrosis.

## HDACs and diabetic kidney disease

Diabetes affects over 451 million people in the world and diabetic nephropathy (DN, kidney disease in diabetes) is the most common cause of CKD. End-stage kidney disease (ESKD) is the last stage of CKD. This is when both kidneys can no longer support the body's needs for day-to-day life. The most common causes of ESRD in the United States are diabetes and high blood pressure [47]. Progressive accumulation of ECM in glomerular mesangium and tubulointerstitium is the hallmark of DN. A number of preclinical studies have demonstrated the efficacy of HDAC inhibition in experimental models of diabetic kidney disease [48, 49]. The early studies reported an increase of HDAC2 activity in streptozotocin (STZ)-induced diabetic kidneys and rat kidney proximal tubular epithelia cells (NRK-52E) exposed to TGF-β1 [50]. siRNA knockdown of HDAC2 reduced the expression of fibronectin and α-SMA in NRK-52E cells. TSA treatment decreased the expression of ECM components and prevented epithelial-mesenchymal-transition (EMT). Valproic acid (VPA) (another HDACI/II inhibitor) or SK704 (selective class I HDAC inhibitor) were observed to have similar effects on NRK-52E cells, which supports the essential role of class I HDACs in the development of diabetic kidney disease [50].

Endoplasmic reticulum (ER) stress caused by reactive oxygen species (ROS) is associated with DN development [51]. Epidermal growth factor receptor (EGFR) mediates oxidative stress and EGFR activation was implicated in diabetic mice. EGFR/AKT/ROS/ER stress signaling plays an essential role in DN progression and inhibiting EGFR may serve as a potential therapeutic strategy in DN [51]. Studies showed that treatment of diabetic rats with vorinostat for 4 weeks significantly decreased EGFR level and repressed kidney growth and glomerular hypertrophy, indicating that HDACs may play role in early DN via EGFR activation [48].

The podocytes are terminally differentiated epithelia cells and present an important component of the kidney filtration barrier [52]. Podocyte damage accelerates the progression of DN through the loss of kidney filtration barrier integrity, resulting in the escape of proteins to the urine (proteinuria) [52]. With database analysis, higher HDAC1 and HDAC2 activity were detected in microarray from glomeruli of proteinuric mice (Inoue et al. 2019). Recently studies demonstrated the important role of podocyte HDAC activity in the regulation of murine and human glomerular disease, and strongly suggest that inhibition of HDAC1 and HDAC2 activities may suppress the progression of human proteinuric kidney disease. Administration of VPA (a class I HDAC inhibitor, FDA-approved drug) and suberanilohydroxamic acid (SAHA) alleviated proteinuria and reduced development to glomerulosclerosis in many rodent glomerular injury models. Podocye-specific HDAC1 and HDAC2 ablation mice were resistant to progressive glomerulosclerosis. In addition, longitudinal analysis of 120,000 participants in the Veteran aging Cohort Study demonstrated a strong protective effect of VPA treatment on the decline of estimated glomerular filtration rate [52].

In summary, kidney disease due to diabetes remains the most common cause of chronic kidney failure, and HDACi provides clinical benefit for the treatment of diabetic kidney disease.

## HDACs and renal carcinoma

HDAC inhibitors have been extensively studied in various cancer types. Renal cell carcinoma (RCC) is the most common kidney cancer and accounts for 2–3% of adult cancers in the United States [53]. Class I HDACs, especially HDAC1, 2, and 3 are highly expressed in RCC, making them interesting targets for therapy [54]. Recent studies showed that HDAC1 and HDAC2 are required for the growth and survival of renal carcinoma cells [55]. HDACis lead to a decrease of E-cadherin (the cell adhesion molecule) and platelet-derived growth factor receptor β (PDGFR, a key driver of RCC metastasis formation) [55]. Clear cell renal cell carcinoma (ccRCC**) **is the most common form of renal cell carcinoma. ccRCC is characterized by the inactivation of the tumor suppressor gene von Hippel Lindau (VHL) [56]. Studies showed that HDAC1 and 6 are highly expressed and modulate cell invasion and migration in ccRCC [56]. Recently, we also detected higher activity of HDAC1 and HDAC2 in Wilms Tumor, a solid cancerous tumor of the kidney that arises from immature kidney cells (Liu et al., unpublished). By targeting both histone and non-histone proteins, HDACs play a critical role in the growth and progression of solid tumors [57]. HDACi has been demonstrated to effectively induce growth arrest, apoptosis, and differentiation of cancer cells, and inhibition of tumor angiogenesis [57, 58].

Accumulating evidence has shown that HDACi inhibit cancer cell proliferation and induce cell cycle arrest [57, 58]. VPA treatment of RCC cells led to G1 arrest by upregulation of p21 [59]. In addition, LBH589 (panobinostat; Farydak®, Novartis Pharms Corp.) induced G2/M arrest of RCC cells by the depletion of aurora A and B and downregulation of Survivin [60], which was specifically mediated through HDAC3 and 6 [60]. Aurora A and B are highly conserved serine/threonine kinases that play important roles during mitosis and meiosis, especially in G_2_-M cell cycle progression. Survivin is a member of the inhibitor of apoptosis (IAP) family, which function to inhibit apoptosis or programmed cell death. G2/M arrest has also been induced by KBH-A145, a γ-lactam-based hydroxamic acid derivative that inhibits HDAC [61]. In human renal cancer cells, KBH-A145 upregulates p21 by inhibiting recruitment of HDAC1 to the p21 promoter [61]. Collectively, these findings indicate that HDAC inhibitors cause cycle arrest in RCC by affecting cell cycle regulators, such as p21, aurora A, and aurora B.

Despite the FDA approval for the treatment of many cancers, the single therapeutic use of HDAC inhibitor (HDACi) has limited therapeutic efficacy against solid tumors [62]. Numerous studies have demonstrated the benefits of combinatorial use of HDACis with other cancer agents for treating RCC [58]. For examples, VPA in combination with low-dosed interferon α, or AEE788 (a receptor tyrosine kinase inhibitor), or RAD001 (an inhibitor of mTOR), is much more effective in inhibiting HDAC activity and cell proliferation in RCC cell [63]. Results of a single-arm phase I/II study show that addition of class 1 HDAC inhibitor entinostat to high dose IL-2 treatment in patients with metastatic ccRCC might be beneficial [64]. The same group also reported that that the recombination of class II HDAC inhibitor vorinostat and the VEGF blocker bevacizumab is relatively well tolerated in single-arm phase I/II clinical trial [65].

Collectively, these studies strongly suggest that HADC inhibition, especially when coupled with additional therapy agents, may provide an effective method for treating RCC.

## Conclusion and outlook

HDACs, especially class I HDACs, have been demonstrated to play a critical role in kidney development [4, 23, 27, 9]. Aberrant expression level and activity of HDACs are closely associated with the pathogenesis and progression of various kidney diseases. Many HDACi have been shown to be very effective in treating kidney diseases. The comprehensive information on the therapeutic effect of histone deacetylase inhibitors on kidney disease was provided in a review paper (Chun, 2018). More studies are necessary to better understand the molecular mechanisms of HDACs in normal kidney formation and kidney diseases. HDACs and HDACi regulate (or alter) gene expression by changing protein acetylation. Currently, the profile of HDAC-modulated or HDCAi-mediated protein acetylation in the kidneys (at physiological or pathological conditions) is not well known. An extensive analysis of global protein lysine acetylation in response to HDAC knockout or HDAC inhibition using proteomic approaches will provide novel insights into the regulation mechanisms of HDACs in kidney development and open new therapeutic avenues for the prevention and treatment of kidney diseases and associated renal-cardiovascular diseases. Numerous studies successfully demonstrated the renoprotective effectors of HDAC inhibitors, however, most of them are pan-HDAC inhibitors. Broad-spectrum HDAC inhibition is more likely to cause nephrotoxicity, and the development of specific HDAC inhibitors is required to improve the clinical outcome and reduce the toxicity. Combinatorial therapy by coupling HDAC inhibitor with additional agents will also be of benefit for the treatment. Furthermore, a comprehensive understanding of the roles of HDACs and HDAC inhibitors will be also of great help for the development of new tools to recapitulate nephrogenesis in regenerative medicine.

## References

[1] Egger G (2004). Epigenetics in human disease and prospects for epigenetic therapy. Nature.

[2] Feil R, Fraga MF (2012). Epigenetics and the environment: emerging patterns and implications. Nat Rev Genet.

[3] Marsit CJ (2015). Influence of environmental exposure on human epigenetic regulation. J Exp Biol.

[4] Liu H (2018). Histone deacetylases 1 and 2 regulate the transcriptional programs of nephron progenitors and renal vesicles. Development.

[5] Schedl A (2007). Renal abnormalities and their developmental origin. Nat Rev Genet.

[6] Aguilera O (2010). Epigenetics and environment: a complex relationship. J Appl Physiol (1985).

[7] Beckerman P, Ko YA, Susztak K (2014). Epigenetics: a new way to look at kidney diseases. Nephrol Dial Transplant.

[8] Wanner N, Bechtel-Walz W (2017). Epigenetics of kidney disease. Cell Tissue Res.

[9] El-Dahr SS, Saifudeen Z (2019). Epigenetic regulation of renal development. Semin Cell Dev Biol.

[10] Berdasco M, Esteller M (2019). Clinical epigenetics: seizing opportunities for translation. Nat Rev Genet.

[11] Timmermann S (2001). Histone acetylation and disease. Cell Mol Life Sci CMLS.

[12] Haberland M, Montgomery RL, Olson EN (2009). The many roles of histone deacetylases in development and physiology: implications for disease and therapy. Nat Rev Genet.

[13] Seto E, Yoshida M (2014). Erasers of histone acetylation: the histone deacetylase enzymes. Cold Spring Harbor Perspect Biol.

[14] Kelly RD, Cowley SM (2013). The physiological roles of histone deacetylase (HDAC) 1 and 2: complex co-stars with multiple leading parts. Biochem Soc Trans.

[15] Vermeulen M (2004). In vitro targeting reveals intrinsic histone tail specificity of the Sin3/histone deacetylase and N-CoR/SMRT corepressor complexes. Mol Cell Biol.

[16] Zhang X (2004). Activation of the growth-differentiation factor 11 gene by the histone deacetylase (HDAC) inhibitor trichostatin A and repression by HDAC3. Mol Cell Biol.

[17] Hayakawa T, Nakayama J (2011). Physiological roles of class I HDAC complex and histone demethylase. J Biomed Biotechnol.

[18] Pflum MK (2001). Histone deacetylase 1 phosphorylation promotes enzymatic activity and complex formation. J Biol Chem.

[19] Tsai SC, Seto E (2002). Regulation of histone deacetylase 2 by protein kinase CK2. J Biol Chem.

[20] Galasinski SC (2002). Phosphatase inhibition leads to histone deacetylases 1 and 2 phosphorylation and disruption of corepressor interactions. J Biol Chem.

[21] Yoon S (2018). PP2A negatively regulates the hypertrophic response by dephosphorylating HDAC2 S394 in the heart. Exp Mol Med.

[22] Montgomery RL (2007). Histone deacetylases 1 and 2 redundantly regulate cardiac morphogenesis, growth, and contractility. Genes Dev.

[23] Chen S (2011). Histone deacetylase (HDAC) activity is critical for embryonic kidney gene expression, growth, and differentiation. J Biol Chem.

[24] Hilliard SA, El-Dahr SS (2016). Epigenetics of renal development and disease. Yale J Biol Med.

[25] Nie L, Young L, Zhang B, Zhao J (2020). Application of histone deacetylase inhibitors in renal interstitial fibrosis. Kidney Dis.

[26] Brunmeir R, Lagger S, Seiser C (2009). Histone deacetylase HDAC1/HDAC2-controlled embryonic development and cell differentiation. Int J Dev Biol.

[27] Chen S (2015). Histone deacetylase 1 and 2 regulate Wnt and p53 pathways in the ureteric bud epithelium. Development.

[28] El-Dahr S, Hilliard S, Saifudeen Z (2017). Regulation of kidney development by the Mdm2/Mdm4-p53 axis. J Mol Cell Biol.

[29] Dressler GR (2009). Advances in early kidney specification, development and patterning. Development.

[30] Kobayashi A (2008). Six2 defines and regulates a multipotent self-renewing nephron progenitor population throughout mammalian kidney development. Cell Stem Cell.

[31] Little MH, McMahon AP (2012). Mammalian kidney development: principles, progress, and projections. Cold Spring Harb Perspect Biol.

[32] Wang L (2012). ASEB: a web server for KAT-specific acetylation site prediction. Nucleic Acids Res.

[33] Brodbeck S, Besenbeck B, Englert C (2004). The transcription factor Six2 activates expression of the Gdnf gene as well as its own promoter. Mech Dev.

[34] Hilliard S (2019). Defining the dynamic chromatin landscape of mouse nephron progenitors. Biol Open.

[35] Kobayashi A (2014). Identification of a multipotent self-renewing stromal progenitor population during mammalian kidney organogenesis. Stem Cell Rep.

[36] Humphreys BD (2010). Fate tracing reveals the pericyte and not epithelial origin of myofibroblasts in kidney fibrosis. Am J Pathol.

[37] Ohmori T (2015). Sall1 in renal stromal progenitors non-cell autonomously restricts the excessive expansion of nephron progenitors. Sci Rep.

[38] Hum S (2014). Ablation of the renal stroma defines its critical role in nephron progenitor and vasculature patterning. PLoS ONE.

[39] Liu N, Zhuang S (2015). Treatment of chronic kidney diseases with histone deacetylase inhibitors. Front Physiol.

[40] Cianciolo Cosentino C (2013). Histone deacetylase inhibitor enhances recovery after AKI. J Am Soc Nephrol JASN.

[41] Pang M (2009). Inhibition of histone deacetylase activity attenuates renal fibroblast activation and interstitial fibrosis in obstructive nephropathy. Am J Physiol Renal Physiol.

[42] Pang M (2011). Histone deacetylase 1/2 mediates proliferation of renal interstitial fibroblasts and expression of cell cycle proteins. J Cell Biochem.

[43] Imai N (2007). Inhibition of histone deacetylase activates side population cells in kidney and partially reverses chronic renal injury. Stem Cells.

[44] Manson SR (2014). HDAC dependent transcriptional repression of Bmp-7 potentiates TGF-beta mediated renal fibrosis in obstructive uropathy. J Urol.

[45] Liu N (2013). Blocking the class I histone deacetylase ameliorates renal fibrosis and inhibits renal fibroblast activation via modulating TGF-beta and EGFR signaling. PLoS ONE.

[46] Yang M (2019). Inhibition of class I HDACs attenuates renal interstitial fibrosis in a murine model. Pharmacol Res.

[47] Hadden MJ, Advani A (2018). Histone deacetylase inhibitors and diabetic kidney disease. Int J Mol Sci.

[48] Gilbert RE (2011). Histone deacetylase inhibition attenuates diabetes-associated kidney growth: potential role for epigenetic modification of the epidermal growth factor receptor. Kidney Int.

[49] Advani A (2011). Long-term administration of the histone deacetylase inhibitor vorinostat attenuates renal injury in experimental diabetes through an endothelial nitric oxide synthase-dependent mechanism. Am J Pathol.

[50] Noh H (2009). Histone deacetylase-2 is a key regulator of diabetes- and transforming growth factor-beta1-induced renal injury. Am J Physiol Renal Physiol.

[51] Xu Z (2017). EGFR inhibition attenuates diabetic nephropathy through decreasing ROS and endoplasmic reticulum stress. Oncotarget.

[52] Inoue K (2019). Podocyte histone deacetylase activity regulates murine and human glomerular diseases. J Clin Investig.

[53] Jacobsohn KM, Wood CG (2006). Adjuvant therapy for renal cell carcinoma. Semin Oncol.

[54] Fritzsche FR (2008). Class I histone deacetylases 1, 2 and 3 are highly expressed in renal cell cancer. BMC Cancer.

[55] Kiweler N (2018). The histone deacetylases HDAC1 and HDAC2 are required for the growth and survival of renal carcinoma cells. Arch Toxicol.

[56] Ramakrishnan S (2016). HDAC 1 and 6 modulate cell invasion and migration in clear cell renal cell carcinoma. BMC Cancer.

[57] Marks PA (2010). Histone deacetylase inhibitors: a chemical genetics approach to understanding cellular functions. Biochem Biophys Acta.

[58] Chun P (2018). Therapeutic effects of histone deacetylase inhibitors on kidney disease. Arch Pharmacal Res.

[59] Jones J (2009). The histone deacetylase inhibitor valproic acid alters growth properties of renal cell carcinoma in vitro and in vivo. J Cell Mol Med.

[60] Cha TL (2009). Dual degradation of aurora A and B kinases by the histone deacetylase inhibitor LBH589 induces G2-M arrest and apoptosis of renal cancer cells. Clin Cancer Res.

[61] Kwon HK (2009). A novel gamma-lactam-based histone deacetylase inhibitor potently inhibits the growth of human breast and renal cancer cells. Biol Pharm Bull.

[62] Suraweera A, O'Byrne KJ, Richard DJ (2018). Combination therapy with histone deacetylase inhibitors (HDACi) for the treatment of cancer: achieving the full therapeutic potential of HDACi. Front Oncology.

[63] Juengel E (2012). Acetylation of histone H3 prevents resistance development caused by chronic mTOR inhibition in renal cell carcinoma cells. Cancer Lett.

[64] Pili R (2017). Immunomodulation by entinostat in renal cell carcinoma patients receiving high-dose interleukin 2: a multicenter, single-arm, phase I/II Trial (NCI-CTEP#7870). Clin Cancer Res.

[65] Pili R (2017). Combination of the histone deacetylase inhibitor vorinostat with bevacizumab in patients with clear-cell renal cell carcinoma: a multicentre, single-arm phase I/II clinical trial. Br J Cancer.

